# Osmotic Edema Rapidly Increases Neuronal Excitability Through Activation of NMDA Receptor-Dependent Slow Inward Currents in Juvenile and Adult Hippocampus

**DOI:** 10.1177/1759091415605115

**Published:** 2015-09-30

**Authors:** Kelli Lauderdale, Thomas Murphy, Tina Tung, David Davila, Devin K. Binder, Todd A. Fiacco

**Affiliations:** 1 Department of Cell Biology and Neuroscience, Riverside, CA, USA; 2 Center for Glial-Neuronal Interactions, University of California Riverside, CA, USA; 3 Division of Biomedical Sciences, UC Riverside School of Medicine, CA, USA

**Keywords:** CA1 pyramidal neuron, bursting activity, action potential, NMDA receptor, extracellular space, epilepsy, seizure, glutamate, slow inward current, extrasynaptic, cell swelling, volume regulated anion channel, hypoosmolar

## Abstract

Cellular edema (cell swelling) is a principal component of numerous brain disorders including ischemia, cortical spreading depression, hyponatremia, and epilepsy. Cellular edema increases seizure-like activity in vitro and in vivo, largely through nonsynaptic mechanisms attributable to reduction of the extracellular space. However, the types of excitability changes occurring in individual neurons during the acute phase of cell volume increase remain unclear. Using whole-cell patch clamp techniques, we report that one of the first effects of osmotic edema on excitability of CA1 pyramidal cells is the generation of slow inward currents (SICs), which initiate after approximately 1 min. Frequency of SICs increased as osmolarity decreased in a dose-dependent manner. Imaging of real-time volume changes in astrocytes revealed that neuronal SICs occurred while astrocytes were still in the process of swelling. SICs evoked by cell swelling were mainly nonsynaptic in origin and NMDA receptor-dependent. To better understand the relationship between SICs and changes in neuronal excitability, recordings were performed in increasingly physiological conditions. In the absence of any added pharmacological reagents or imposed voltage clamp, osmotic edema induced excitatory postsynaptic potentials and burst firing over the same timecourse as SICs. Like SICs, action potentials were blocked by NMDAR antagonists. Effects were more pronounced in adult (8–20 weeks old) compared with juvenile (P15–P21) mice. Together, our results indicate that cell swelling triggered by reduced osmolarity rapidly increases neuronal excitability through activation of NMDA receptors. Our findings have important implications for understanding nonsynaptic mechanisms of epilepsy in relation to cell swelling and reduction of the extracellular space.

## Introduction

*These authors contributed equally to this workCerebral edema affects millions of people worldwide and is a complication associated with a wide range of diseases, disorders, and conditions of the nervous system including traumatic brain injury, stroke, cardiac arrest, autism, and epilepsy. Cellular (or “cytotoxic”) edema is strongly linked to brain excitability, which has historically been studied in the context of seizure (Schwartzkroin et al., 1998). In epilepsy, cell swelling precedes initiation of seizure-like discharges in vitro and in vivo (Traynelis and Dingledine, 1989; Binder et al., 2004). Cellular edema induced by reduction of solution osmolarity can itself trigger seizure-like activity, while at lower doses it increases the amplitude and frequency of synchronous bursting (Andrew et al., 1989; Dudek et al., 1990; Ballyk et al., 1991; Saly and Andrew, 1993; Kilb et al., 2006). Inhibition of cell swelling by increasing solution osmolarity prevents initiation of ictal discharges and stops spontaneously recurring seizures in progress (Andrew et al., 1989; Traynelis and Dingledine, 1989; Haglund and Hochman, 2005).

The relationship between hypoosmolarity and seizures prompted studies to understand the mechanisms underlying the effects of lowered osmolarity on neuronal excitability in brain slices. In pioneering work, it was found that evoked population spikes (PS), field potentials, and whole-cell evoked excitatory postsynaptic currents (EPSCs) were enhanced in hypoosmolar artificial cerebrospinal fluid (hACSF; Andrew et al., 1989; Ballyk et al., 1991; Chebabo et al., 1995a; Huang et al., 1997). Excitability of individual CA1 pyramidal neurons remained stable, as gauged by resting membrane potential, cell input resistance and action potential (AP) threshold (Andrew et al., 1989; Ballyk et al., 1991). It was concluded that a combination of nonsynaptic, electrical field effects, and enhanced chemical synaptic transmission increased the excitability of the population of CA1 pyramidal neurons (Ballyk et al., 1991; Huang et al., 1997). Both effects can be explained by a concomitant reduction of the extracellular space (ECS): During cell swelling, increased tissue resistance enhances the voltage across neuronal membranes (Andrew et al., 1989), while also elevating the concentration of ambient neurotransmitter molecules in the vicinity of ligand-gated ion channels (Huang et al., 1997). A limitation of these studies was that effects on spontaneous neuronal activity were not measured, and evoked recordings were performed only after several minutes in reduced osmolarity conditions.

A more recently described form of excitability observed in pyramidal neurons in several brain areas are slow inward currents (SICs; Fellin et al., 2004; Kozlov et al., 2006; Fiacco et al., 2007). SICs are compelling because they can be evoked when synaptic transmission is blocked, they are NMDA receptor-dependent, they can be of very large amplitude, and they have slow kinetics, suggesting slow diffusion of glutamate. Because of their nonsynaptic origin and slow kinetics, together with the possible role of extrasynaptic NMDA receptors in brain pathology and epilepsy (Dingledine et al., 1986; Traynelis and Dingledine, 1988; Hardingham and Bading, 2010), SICs fit well with both nonsynaptic and neurochemical properties of seizure-like discharges. SICs have been reported to be synchronized among small groups of pyramidal neurons (Angulo et al., 2004), suggesting that they may be a type of focal epileptiform activity (Wetherington et al., 2008). Although SICs were first reported to be driven by Ca^2+^-dependent glutamate release from astrocytes, subsequent studies have pointed to a crucial role of solution osmolarity and cellular swelling to the generation of SICs (Kozlov et al., 2006; Fiacco et al., 2007). Overall, the study of SICs has been very limited. The relationship between hypoosmolarity, SICs, and changes in neuronal excitability in both the juvenile and adult brain is poorly understood.

In this study, we set out to extend previous work on the effects of osmolarity on evoked neuronal events by determining if there are acute changes in spontaneous neuronal excitability upon switch to hypoosmolar ACSF (hACSF). By continuously recording spontaneous whole-cell currents in CA1 pyramidal cells in acute mouse hippocampal slices, we found that switching to hACSF evoked SICs within about 1 min of application. Most large amplitude SICs were evoked within the first 3 min of hACSF application and then tapered off. As in previous studies, SICs were evoked when synaptic transmission was blocked and were NMDA receptor-dependent. SICs were accompanied by a hyperpolarizing shift in baseline current, indicating direct effects of osmolarity on the resting potential of individual neurons which was confirmed in subsequent current-clamp experiments. Because SICs have traditionally been recorded in the presence of several pharmacological reagents, it is not clear what role SICs have, if any, on neuronal excitability under more physiological conditions. In the absence of NBQX and D-serine, at physiological temperature and with Mg^2+^ present in the bath, hACSF evoked EPSPs and APs in individual CA1 pyramidal cells over the same timecourse as SICs. APs evoked by hACSF application exhibited significantly increased bursting characteristics compared with spontaneous APs occurring prior to hACSF application. Neuronal excitability was rapidly increased by hACSF in hippocampal slices from both juvenile and adult mice, with effects in adults more pronounced than in juveniles. Collectively, our findings point to a critical role for SICs in elevating spontaneous neuronal excitability over a rapid timecourse in a variety of conditions and provide insight into increased excitability associated with cerebral edema and underlying mechanisms of epilepsy.

## Materials and Methods

All animals and protocols used in these experiments were approved by the Institutional Animal Care and Use Committee (IACUC) of the University of California, Riverside and followed the approved protocols established by the American Veterinary Medical Association.

### Slice Preparation

Hippocampal slices were prepared from wild-type C57Bl/6J mice as previously described (Xie et al., 2014). Briefly, juvenile (P15-21) mice were deeply anesthetized under isoflurane and decapitated. Brains were removed into ice-cold slicing buffer containing (in mM): 125 NaCl, 2.5 KCl, 3.8 MgCl_2_, 1.25 NaH_2_PO_4_, 26 NaHCO_3_, 25 glucose, 1.3 ascorbic acid, and 3.5 MOPS, bubbled with 5% CO_2_ + 95% O_2_. Parasagittal hippocampal slices (350 µm) were prepared using a Leica VT 1200S Vibratome (Bannockburn, IL) and subsequently incubated for 45 min at 35℃ in standard ACSF, which contained the following (in mM): 125 NaCl, 2.5 KCl, 2.5 CaCl_2_, 1.3 MgCl_2_, 1.25 NaH_2_PO_4_, 26.0 NaHCO_3_, and 15 glucose, oxygenated with 5% CO_2_ + 95% O_2_, (∼300–303 mOsm). After recovery, slices were allowed to cool to room temperature for a minimum of 15 min prior to electrophysiological recording.

Adult hippocampal slices were prepared from male 2 to 5-month-old mice as described above, using a modified slicing buffer containing the following (in mM): 87 NaCl, 75 sucrose, 2.5 KCl, 0.5 CaCl_2_, 7 MgCl_2_, 1.25 NaH_2_PO_4_, 25 NaHCO_3_, 10 glucose, 1.3 ascorbic acid, 3.5 MOPS, and 100 µM kynurenic acid, bubbled with 5% CO_2_ + 95% O_2_. Adult slicing buffer was partially frozen to achieve a “slushy” consistency for slice preparation, as we have observed a drastic improvement in adult slice health via this method. Slices were then incubated in adult slicing buffer at 35℃ for 45 min. Following recovery, slices were allowed to cool to room temperature for 15 min before being transferred to room temperature standard ACSF and equilibrating for an additional 20 to 30 min prior to recording.

### Electrophysiology

Whole-cell patch clamp recordings were made using a Multiclamp 700B amplifier and Multiclamp Commander software, with either a DigiData 1550 or DigiData 1440A digitizer, and Clampex 10.2–10.4 software (Molecular Devices, Sunnyvale, CA). Patch pipettes were pulled from borosilicate glass capillaries (World Precision Instruments), to resistances of 3.8 to 5.9 MΩ when filled with neuronal internal solution containing the following (in mM): 140 K-gluconate, 4 MgCl_2_, 0.4 EGTA, 4 Mg-ATP, 0.2 Na-GTP, 10 HEPES, and 10 phosphocreatine, pH 7.3 with KOH. Neurons were identified by their location in the pyramidal cell layer, morphological characteristics, resting membrane potential, and characteristic voltage-gated sodium and potassium currents using a voltage step protocol (Fiacco and McCarthy, 2004). Continuous recordings for both voltage- and current-clamp experiments were low-pass filtered at 2 kHz and digitized at 5 kHz. All experiments were conducted at room temperature (23℃–26℃) unless otherwise stated.

For volume imaging experiments, stratum radiatum astrocytes were whole-cell patch clamped using patch pipettes pulled to a resistance of 5.5 to 8.9 MΩ. The internal solution contained the following (in mM): 130 K-gluconate, 4 MgCl_2_, 10 HEPES, 10 glucose, 1.185 Mg-ATP, 10.55 phosphocreatine, and 0.1315 mg/ml creatine phosphokinase, pH 7.3 by KOH. Astrocyte internal solution also contained 100 to 200 µM Alexa Fluor 488 dextran or Oregon Green 488 dextran, MW = 10,000 (Life Technologies). High molecular weight dextrans were used to avoid dye leakage into adjacent astrocytes, which tends to dim the cell over time and impair volume analysis. Stratum radiatum astrocytes were identified first by morphology and location, and second by their characteristic low input resistance, low resting potential (−78.0 ± 1.2 mV), and lack of voltage-gated conductances. Upon attaining whole-cell configuration, astrocytes were voltage-clamped approximately 10 mV more negative than their resting potential to assist with loading of the negatively charged fluorescent indicators. Fluorescent dye was allowed to dialyze into the cell for ≤10 min and effective loading was verified using brief, low-power confocal scans. Once loading was deemed sufficient, the pipette was carefully withdrawn. A smooth, stable off-cell and formation of an outside-out patch were considered indicative of minimal damage to the membrane; any cells not fitting these criteria were discarded.

### Neuronal Excitability in hACSF

For experimental conditions, “normosmolar” ACSF (nACSF) was prepared the same as standard ACSF but made Mg^2+^-free by omission of MgCl_2_ (∼300 mOsm). nACSF also contained 1 µM tetrodotoxin (TTX) (Abcam) in voltage-clamp experiments, plus 10 µM of the selective AMPA or Kainate receptor antagonist NBQX (Abcam) in all experiments, unless indicated otherwise. In some experiments, the NMDA receptor co-agonist D-serine (10 µM; Ascent Scientific), the NMDA receptor antagonist DL-AP5 (50 µM; Abcam), or the NR2B subunit-containing NMDA receptor blocker Ro 25-6981 (1 µM; Tocris) were included to assess NMDA receptor function upon switch to reduced osmolarity ACSF. hACSF was prepared by dilution of nACSF with deionized water (%v/v) to 5% (∼285 mOsm), 10% (∼270 mOsm), 17% (∼250 mOsm), or 40% (∼180 mOsm) hypoosmolar compared with baseline, as measured using a Vapro 5520 vapor pressure osmometer (Wescor). In general, hippocampal CA1 pyramidal neurons were patch-clamped first in standard ACSF. Upon attaining whole-cell configuration, nACSF was perfused for 10 min to remove Mg^2+^ block from NMDA receptors and obtain a baseline recording of excitatory neuronal activity. This was followed by a 5-min application of hACSF followed by an equal length wash period in nACSF. These latter two steps were repeated for experiments in D-serine or Ro 25-6981 to allow for within-cell comparisons of pharmacological effects. In voltage-clamp experiments, neurons were clamped at −70 mV. With our K-gluconate-based internal solution, this holding potential equals the calculated reversal potential for Cl^−^ and, therefore, effectively eliminates contribution of fast IPSPs to the recorded currents. To avoid potential confounds from litter-specific anomalies, no more than three slices or three mice per litter were used per group for any experiment. Only one neuron was recorded per slice, and experimental conditions (e.g., 17% or 40% hACSF) were alternated between slices. Access resistance was checked before and after each segment of a recording (baseline, hACSF, and wash). Neurons in which access changed more than 20% during any recording segment were excluded from analysis.

### Volume Imaging

Astrocytes and were visualized using an Olympus BX61 WI upright microscope equipped with UMPLFLN 10× (N.A. 0.3) and LUMFLN 60× (N.A. 1.1) water-immersion objectives and DIC optics. Following dye loading and outside-out patch formation (see earlier), the bath solution was switched to nACSF (Mg^2+^-free ACSF) containing 1 µM TTX and 10 µM NBQX for 10 min prior to taking a baseline image of the astrocyte soma. After measuring the baseline volume, hACSF + TTX + NBQX was applied for 5 min, during which additional images were taken at the start of each minute. Once the last image in hACSF was acquired, the solution was switched back to nACSF for an additional 5 min. A final image was taken at the end of this wash period.

Alexa Fluor 488 and OGB-1 dextran dyes were imaged using a 488 nm argon laser and detected with a 503 to 548 nm bandpass filter. Laser power was held at 1.5% or below to limit any possible photobleaching or phototoxicity. Obtaining single images through the same plane of section in a cell has proven difficult owing to the rapid *z*-axis movement of a swelling slice and in general does not fully capture the borders of a typical astrocyte soma. Therefore, *z*-stacks were taken through the soma at each time point, with an optical slice interval of 1.00 µm. This ensured that all edges of the astrocyte soma would be visible, regardless of slice swelling or cell orientation. To maintain both high resolution and fast scan speed, images were acquired using a zoom factor of 3.5× and were clipped as close to the soma as reasonably possible.

### Analysis and Statistics

**Figure 3.**
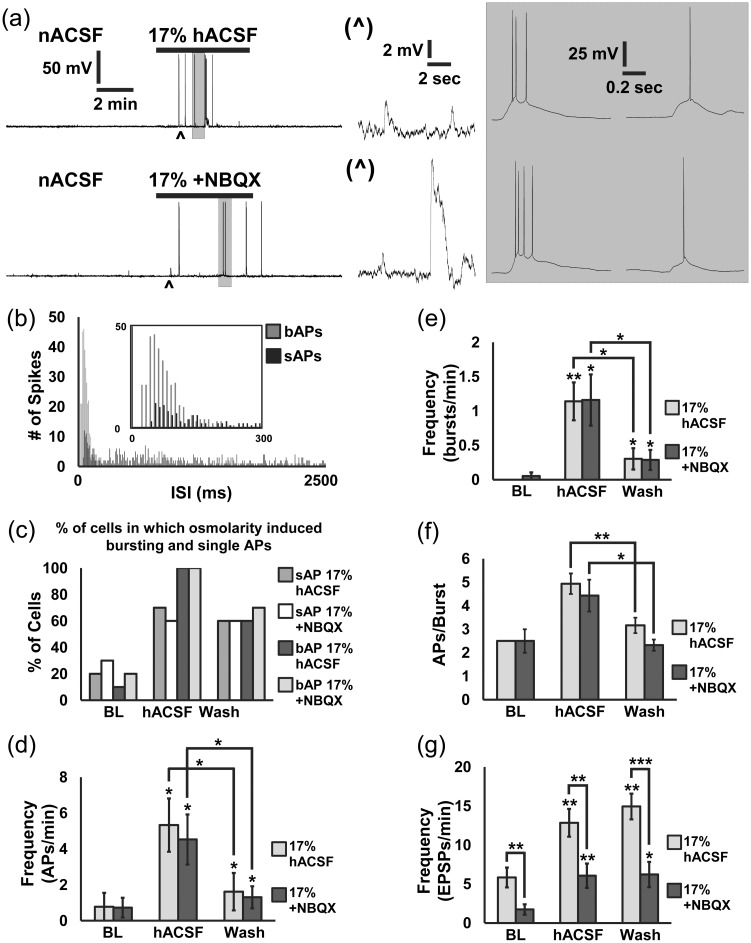
Seventeen percent of hACSF evokes neuronal action potentials and increased bursting activity that is independent of AMPA receptor activation. (a) Current-clamp recording of neuronal membrane potential in nACSF (baseline period) followed by 17% hACSF without NBQX (upper traces) and in 10 µM NBQX (lower traces). Asterisks mark expanded traces of EPSPs (middle panels), while shaded boxes indicate expanded traces of single APs and bursting activity (right panels). Seventeen percent of hACSF evoked APs independent of AMPA receptors. (b) Plotting interspike interval into 10 ms bins revealed that most bursting activity correlated with the highest frequency of APs, but that single APs could also occur at high frequency and were more uniform in their distribution. (c) The percentage of cells exhibiting single and bursting APs was much greater in the hACSF condition compared with baseline. Bursting occurred in 100% of CA1 pyramidal neurons in hACSF compared with only 10% to 20% in nACSF. The percentage of cells in which reduced osmolarity induced bursting and single APs was almost the same in 10 µM NBQX, indicating that AMPA receptors did not participate in the effect. (d, e) While spontaneous APs and bursting activity were infrequent in control conditions, 17% hACSF and 17% hACSF + NBQX evoked frequent APs and bursts. Frequency of APs and bursts declined significantly during the wash period but were still significantly elevated compared with baseline. (f) The number of action potentials within a burst was higher on average in 17% hACSF compared with baseline and wash periods. (g) NBQX reduced by approximately half the frequency of subthreshold EPSPs. Although EPSPs were partially blocked by NBQX, this did not affect the ability of 17% hACSF to evoke single and bursting APs in CA1 pyramidal cells. (*p < .05, **p < .01, and ***p < .001) *n* = 8 to 10 cells per group.

Electrophysiological recordings of neuronal currents or membrane potential were analyzed using Clampfit 10.2–10.4 software (Molecular Devices, Sunnyvale, CA). Baselines were manually adjusted prior to event detection in order to offset the hyperpolarizing shift (outward current in voltage-clamp) observed in hACSF. All events were detected using threshold searches with parameters adjusted accordingly. SICs were detected semi-automatically as inward currents ≥20 pA, with rise times slower than 10 ms (Angulo et al., 2004; Fellin et al., 2004, 2006). Events detected twice were manually rejected. Excitatory postsynaptic potentials (EPSPs) were detected semi-automatically as events ≥2 mV, and APs were detected automatically as positive deflections ≥65 mV above baseline. APs were classified as bursts (bAPs) according to the following criteria: (a) Two or more spikes must ride atop a longer, plateau-like EPSP (Kim and Connors, 1993; Helmchen et al., 1999; Larkum and Zhu, 2002) and (b) Interspike intervals (see Figure 3(b)) for spikes in a burst must fall within 2 standard error lengths of the interspike interval distribution for all cells (Cocatre-Zilgien and Delcomyn, 1992).

Statistical analysis of event amplitude, frequency, and kinetics was performed using IBM SPSS Statistics 22. Split plot repeated measures ANOVAs were used to account for between-group factors (e.g., hACSF dose) as well as within-cell factors (baseline, hACSF, or wash) in each experiment and followed by planned comparisons using the Student’s *t*-test. Holm-Bonferroni or Tukey’s honest significant difference correction for multiple comparisons was used to adjust α as necessary. Statistical significance for the *p* values is as follows: (*p < .05), (**p < .01), and (***p < .001). *N* = 8 to 10 cells for all experiments, with an equal number of cells per group for parametric comparison.

Analysis of astrocyte volume was performed off-line using Fiji/ImageJ. Image stacks at each time point were *z*-adjusted to correct for cell “drift” caused by slice swelling during hypoosmolar treatment. Stacks were then concatenated into an x-y-z-t hyperstack and filtered to remove noise (median filter, 2 pixel diameter). Next, a max-intensity projection was taken through the *z*-plane to produce a 2D time series, where each image showed the full extent of the soma at that time point. After registration and background subtraction, the finalized time series was binarized using the “mean” thresholding algorithm. An elliptical region of interest (ROI) was drawn to encompass the soma as closely as possible throughout the time series, and the resulting thresholded area encompassed by the ROI was quantified. Soma area was normalized to baseline for each astrocyte and measured as percent change over baseline for each time point in hACSF and wash. Percent changes in astrocyte volume were analyzed by repeated measures ANOVA with posthoc tests and adjusted for multiple comparisons by Bonferroni correction (*p < .05, ***p < .001). *N* = 7 to 8 cells per condition.

## Results

### Hypoosmolar Challenge Evokes Large Excitatory SICs in Neurons That Correlate With Real-Time Volume Changes in Astrocytes

**Figure 1.**
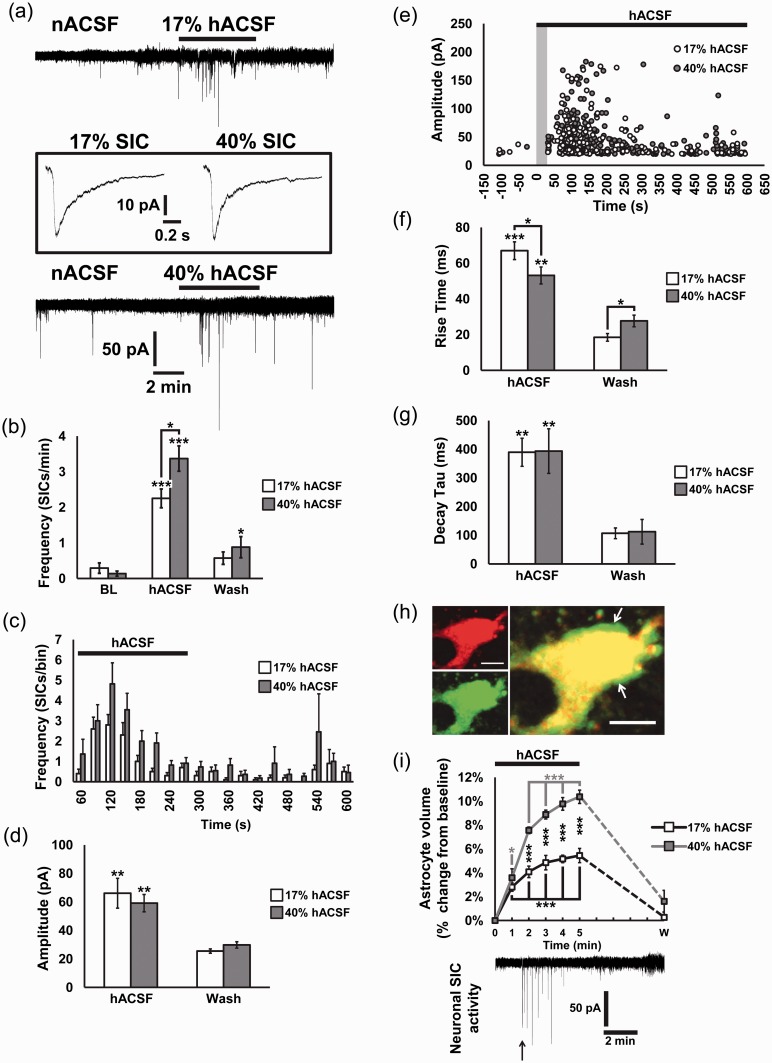
Large neuronal SICs are evoked by hACSF and occur during astrocyte volume increases. (a) Whole-cell voltage clamp recordings of neuronal excitatory currents in TTX (1 µM) and NBQX (10 µM) in normosmolar ACSF (nACSF; baseline condition) and in 17% or 40% hypoosmolar ACSF (hACSF). Switch from nACSF to hACSF evoked SICs in neurons within approximately 60 s on average. Note that most SICs were evoked within the first few minutes of hACSF application. SICs evoked during hACSF application are averaged (continued) **Figure 1**. Continued. and shown in the inset for each condition. (b) Summary histogram of SIC frequency during hACSF application compared with the baseline period and wash. SICs continued to occur during the wash period but at a much lower rate. Frequency of SICs was greater in 40% compared with 17% hACSF. (c) Frequency of SICs analyzed in 30 s bins over the hACSF and wash periods indicates that most SICs occur in the early part of the hACSF application, peak at about 120 s and gradually decline. SICs occurring during the wash period were more variable. (d) There was no difference in the amplitude of SICs on average between 17% and 40% hACSF. Amplitudes of SICs in both conditions were significantly larger compared with SICs occurring over the wash period. (e) Scatter plot of amplitudes of all SICs occurring during the hACSF and wash periods reveals that most large amplitude SICs occurred during osmotic challenge, although overall, amplitudes were highly variable. (f, g) SICs occurring during hACSF application had very slow kinetics, with rise times of 50 to 60 ms and decay taus around 380 ms. These kinetics were significantly slower compared with SICs occurring over the wash period. (h) Baseline images of astrocytes loaded with fluorescent dextran, after 10 min in nACSF (upper left panel) and 5 min in 40% hACSF (lower left panel). Right panel is overlay, with arrows indicating regions of the astrocyte soma with expanded volume. Images are pseudocolored to differentiate between nACSF and hACSF conditions. (i) Volume changes in astrocytes quantified as percent change from baseline in 1-min intervals. Significant increase in astrocyte volume occurred within 1 min in both 17% and 40% hACSF. Astrocyte volume continued to rise throughout the 5 min application of hACSF and recovered to baseline after a 5-min wash period in nACSF. Note that astrocyte volume increases initiated prior to SICs evoked in adjacent neurons, and that SICs occur *while astrocyte volume is increasing*. (*p < .05, **p < .01, and ***p < .001) Neurons, *n* = 10 cells per group; Astrocytes, *n* = 7 to 8 cells per group. Scale bars, 5 µm.

Previous studies have found a contribution of hypoosmolarity to the generation and strength of seizure-like activity in hippocampal slices (Andrew et al., 1989; Ballyk et al., 1991; Saly and Andrew, 1993). However, these studies examined the effects of osmotic edema only on evoked neuronal activity 10 to 20 min after osmotic challenge. To complement this earlier work, we examined the immediate timecourse of effects of osmotic edema on spontaneous neuronal excitability and real-time astrocyte volume changes in the hippocampus. As a first experiment, we measured the effects of acute osmotic edema on neuronal excitability by recording putative NMDA receptor currents. NMDA receptors have been shown to play a prominent role in seizure activity in the hippocampus during cell swelling and reduction of the ECS (Traynelis and Dingledine, 1988, 1989). Hippocampal slices were exposed to Mg^2+^-free mild (17%) or moderate (40%) hACSF while recording excitatory currents in CA1 pyramidal neurons in the presence of the AMPA receptor antagonist NBQX (10 µM). Chloride conductances were prevented from contributing to the recorded currents by voltage-clamping the neurons at the chloride reversal potential (see Materials and Methods section). Spontaneous EPSCs were recorded over a 10-min baseline period in nACSF prior to a 5 min. application of mild or moderate hACSF. Following the hACSF application, the solution was switched back to nACSF for a 5 min “wash” period. Application of 17% or 40% hACSF produced a positive shift in holding current (27.92 ± 3.92 pA in 17%; 42.17 ± 2.34 pA in 40%) and evoked large excitatory inward currents with slow kinetics within 1 min of hACSF application (Figure 1(a)). These SICs were identified and distinguished from the remaining synaptic mEPSCs if they had rise times slower than 10 ms and amplitudes ≥20 pA (Angulo et al., 2004; Fellin et al., 2004, 2006). Occurrence of SICs over the baseline period was either nonexistent (5/10 cells in 17% hACSF, 7/10 cells in 40% hACSF) or extremely infrequent (0.29 ± 0.15 SICs/min in 17% hACSF, 0.14 ± 0.07 SICs/min in 40% hACSF; Figure 1(b)). Frequency of SICs peaked approximately 2 min into hACSF treatment regardless of dose and correspondingly diminished to baseline approximately 2 to 3 min into the wash period (Figure 1(b) and (c)). Frequency of SICs evoked in 40% hACSF remained marginally but significantly elevated during the wash period (0.88 ± 0.30 SICs/min, *p* < .05). On average, SICs evoked in hACSF had large amplitudes (17% hACSF: 66.15 ± 10.49 pA; 40% hACSF: 59.19 ± 6.09 pA), which declined significantly over the wash period (17% wash: 25.53 ± 1.50 pA; 40% wash: 29.82 ± 2.26 pA; Figure 1(d) and (e)). Kinetics of SICs evoked in hACSF were significantly slower than SICs occurring over the baseline or wash periods (Figure 1(f) and (g)). Rise times of SICs in 40% hACSF were significantly faster compared with 17% but were slower during the wash. These findings suggest that SICs are one of the first events occurring during acute osmotic edema to increase neuronal excitability.

The slow kinetics of SICs observed in hACSF support the notion that hACSF slowed glutamate diffusion through a compressed ECS. If this is the case, we would expect to observe significant cellular edema within 2 min of hACSF application (when SIC frequency peaks). Astrocytes in particular are capable of swelling during long periods of reduced osmolarity and have been reported to swell selectively in such conditions (Andrew et al., 2007; Risher et al., 2009). Examination of astrocyte volume in our conditions revealed that hACSF induced rapid swelling of astrocytes within the first minute of application (Figure 1(h) and (i); 17% hACSF, *p* < .001; 40%, *p* < .05). Astrocyte volume increased most rapidly over the first 1 to 2 min after exposure to hACSF. This timecourse closely mirrored the frequency and amplitude distributions of SICs (Figure 1(c) and (e)), suggesting that astrocyte swelling may be important for reduction of the ECS and generation of SICs in the early stages of osmotic edema.

**Figure 2.**
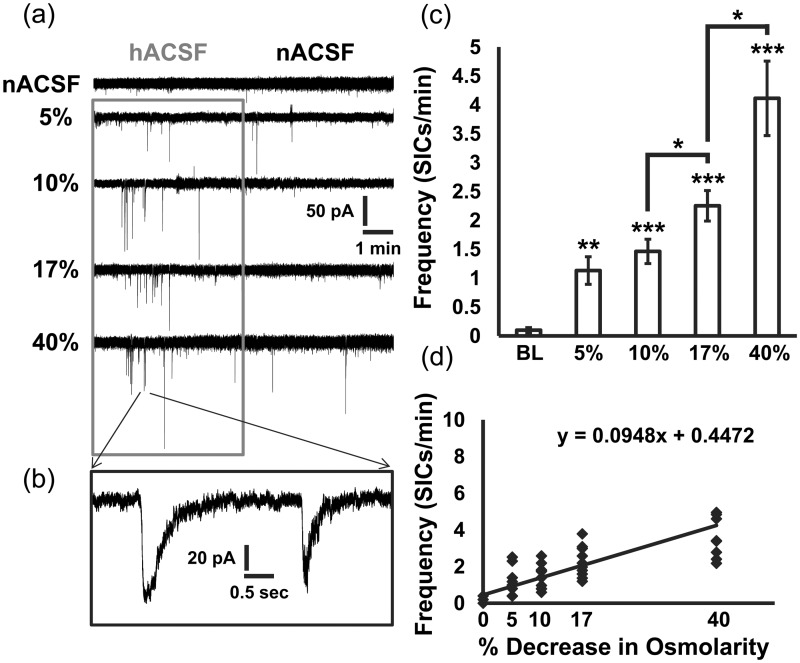
Hypoosmolar ACSF increases neuronal SICs in a dose-dependent manner. (a) Representative recordings of SICs from CA1 pyramidal neurons evoked in 5%, 10%, 17%, and 40% hACSF (gray box; 5 min) followed by a 5-min wash period in normosmolar ACSF. (b) Five-second zoomed-in recording from the 40% hACSF to demonstrate the characteristic size and timecourse of SICs evoked during cell swelling. (c) Frequency of SICs per minute was significantly elevated over baseline during a 5-min application of 5%, 10%, 17%, and 40% hACSF. (d) Scatter plot of frequency of SICs per minute for each cell as osmolarity decreased demonstrates a near linear dose–response relationship between hypoosmolarity and neuronal excitability. (*p < .05, **p < .01, and ***p < .001) *n* = 10 cells per group.

Frequency of SICs was highly sensitive to percent reduction in osmolarity, prompting us to more specifically probe the dose–response relationship of hACSF to SIC generation. Therefore, we recorded the effect of 5%, 10%, 17%, or 40% hACSF on the generation of SICs (Figure 2). SICs were evoked in all conditions and also very rarely occurred spontaneously over the Mg^2+^-free baseline period (Figure 2(a–c)). Frequency of SICs increased significantly even in 5% hACSF compared with baseline (nACSF: 0.10 ± 0.04 SICs/min; 5% hACSF: 1.13 ± 0.24 SICs/min; *p* < .01). Moreover, SICs were significantly more frequent in 17% hACSF compared with 10% hACSF and in 40% hACSF compared with 17% hACSF. These data indicate an approximately linear increase in the generation of SICs with decreasing osmolarity (Figure 2(d)) and with no threshold for initiation, suggesting that even the smallest reductions in osmolarity are potentially sufficient to increase neuronal excitability.

### Osmotic Edema Evokes APs and Excitatory Potentials in CA1 Pyramidal Neurons that Persist When AMPA Receptors Are Blocked and in Physiological Levels of Mg^2+^

In the recordings of neuronal currents described thus far, neurons were voltage-clamped at a holding potential of −70 mV, and synaptic transmission in the slice was suppressed by TTX and an AMPA receptor antagonists. While these conditions are ideal to isolate neuronal SICs, they fall short of physiological conditions in which neuronal membrane potential fluctuates and AMPA receptors influence neuronal excitability. Furthermore, the amplitudes of many SICs seem large enough to depolarize neurons above firing threshold, a possibility we wanted to test by recording membrane voltage. Therefore, we next recorded the effects of mild osmotic edema on APs and EPSPs in CA1 pyramidal neurons (Figure 3). Confirming the positive shift in holding current observed in voltage-clamp recordings, application of 17% hACSF rapidly hyperpolarized the membrane potential of CA1 pyramidal neurons by −3.69 ± 0.51 mV. Despite hyperpolarizing neuronal V_m_, APs, and EPSPs were evoked by 17% hACSF (Figure 3(a), upper traces), as well as in 17% hACSF + NBQX (10 µM; lower traces). Previous work reported that SICs evoked by astrocyte calcium elevations often induced slow bursts of APs (Fellin et al., 2006). Therefore, we subdivided APs into bursts or single APs (bAPs or sAPs, respectively) for our analysis. Bursts were differentiated from single APs as groups of multiple spikes atop a single depolarizing potential (Figure 3(a), gray boxes), generally with interspike intervals shorter than 200 ms (Figure 3(b); see Methods). Similar to our observations of SICs, few neurons generated either sAPs or bAPs in nACSF, but exhibited a dramatic increase in both types of APs during hACSF application (Figure 3(c)). Total AP frequency (Figure 3(d)) was elevated significantly above baseline in 17% hACSF ± NBQX (−NBQX: 5.33 ± 1.49 APs/min; +NBQX: 4.53 ± 1.40 APs/min; *p* < .05), and declined significantly after return to nACSF (−NBQX: 1.62 ± 1.05 APs/min; +NBQX: 1.31 ± 0.62 APs/min; *p* < .05). Of the two patterns of AP activity, bAPs were uniquely enhanced by 17% hACSF application, increasing to 1.14 ± 0.28 bAPs/min in 17% hACSF (*p* < .01) and to 1.16 ± 0.37 bAPs/min in 17% hACSF + NBQX (*p* < .05; Figure 3(e)). Bursting APs dropped back down during the wash period to near baseline levels (−NBQX: 0.30 ± 0.16 bAPs/min; +NBQX: 0.29 ± 0.15 bAPs/min), although neither completely returned to baseline. Reduced frequency of bursts during the wash was accompanied by a significant decrease in the number of APs within each burst (Figure 3(f)). Addition of NBQX did not affect bAP frequency or properties and was only effective in reducing EPSP frequency (Figure 3(g)). Reduction of EPSPs by NBQX is not surprising given the large increase in network activity and incoming APs into the recorded cell. These findings suggest that the generation of APs themselves is largely independent of AMPA receptor activation, while synaptic AMPAR activity increases as a result of the elevated APs evoked by osmotic edema.

**Figure 4.**
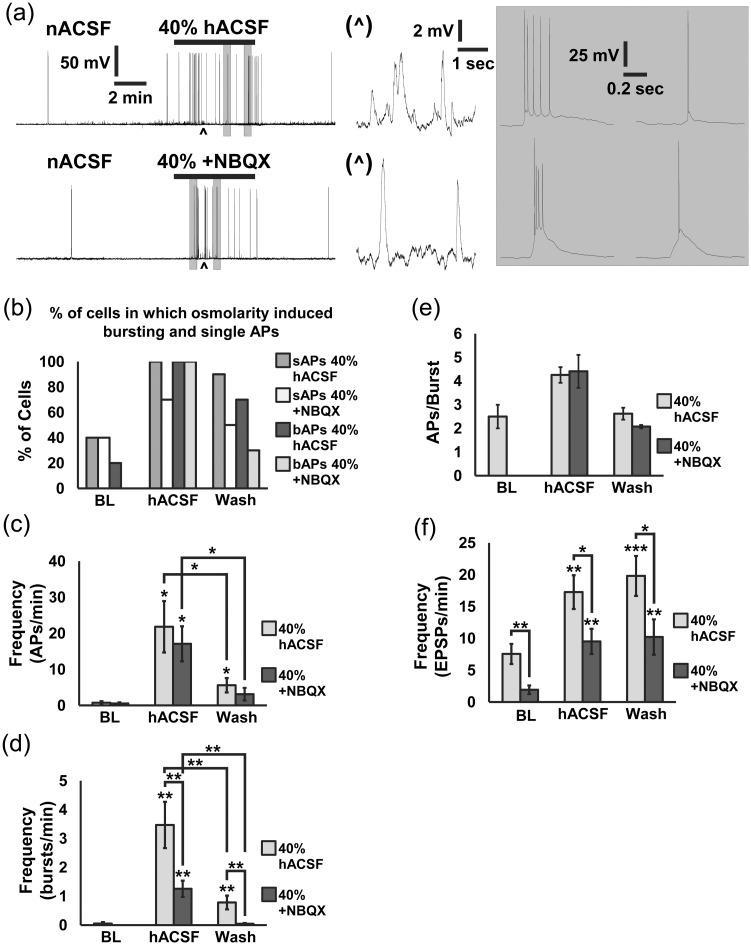
Forty percent of hACSF evokes neuronal action potentials and increased bursting activity that is partly dependent on AMPA receptor activation. (a) Whole cell current-clamp recordings of neuronal action potentials and EPSPs in normosmolar ACSF (baseline) followed by 40% hACSF (upper traces) or 40% hACSF + 10 µM NBQX (lower traces). Carets mark location of expanded traces of EPSPs (middle panels), while shaded boxes mark expanded traces of APs (right panels). Note the combination of single APs and bursting activity evoked by hACSF ± NBQX. (b) Nearly all CA1 pyramidal cells fired APs and displayed bursting activity in hACSF, while less than half the cells fired APs over the baseline period where bursting was rare. The percentage of cells with APs and bursts declined during the wash period. (c, d) Frequency of APs and bursting activity significantly increased during application of 40% hACSF compared with baseline and wash periods. NBQX did not affect the frequency of single APs evoked by hACSF (c), but it did significantly reduce bursting activity (d). (e) The number of APs per burst was higher during hACSF application compared with baseline and wash periods, although the number of cells exhibiting bursts over the baseline and wash periods was too low to run statistics. (f) Frequency of EPSPs was significantly elevated in 40% hACSF and wash periods over baseline and reduced significantly in all conditions by NBQX. The AMPA receptor dependent synaptic activity is not surprising given the frequency of APs occurring in the network onto the recorded cell. (*p < .05, **p < .01, and ***p < .001) *n* = 9 to 10 cells per group.

On the basis of our observations that 40% hACSF evoked a significantly greater frequency of SICs compared with 17% hACSF (Figure 1(b)), we reasoned that APs and EPSPs would also increase in a similar manner and with similar dependence (or lack thereof) on AMPA receptors. We tested this by replicating the above experiments using 40% hACSF, once again in both the presence and absence of NBQX (Figure 4(a)). Application of 40% hACSF caused neurons to hyperpolarize nearly twice as much as 17% hACSF (−7.35 ± 0.91 mV vs. −3.69 mV for 17%). As before, the number of neurons displaying bAPs jumped from less than half at baseline, up to 100% during application of 40% hACSF (Figure 4(b)). This dose of hACSF also increased the number of cells displaying sAPs, reaching 100% participation in the absence of NBQX. The percentage of neurons in which APs were evoked diminished over the wash period. Total AP frequency in 40% hACSF ± NBQX was 3 to 4 times higher than in 17% hACSF (−NBQX: 21.81 ± 7.13 APs/min; +NBQX: 17.09 ± 4.86 APs/min), and once again significantly decreased after return to nACSF (Figure 4(c)). Much more pronounced was the effect of NBQX on bAP activity in the 40% versus the 17% hACSF (Figure 4(d)). Although bursting frequency was significantly higher in 40% hACSF and remained elevated during wash, NBQX inhibited the hACSF-induced increase by more than 50% (+NBQX: 1.26 ± 0.28 bAPs/min; *p* < .01 vs. −NBQX) and also reduced burst frequency to baseline levels after hACSF washout. Interestingly, despite the significant effect of NBQX on bAP frequency in 40% hACSF, NBQX did not alter the number of spikes per burst (Figure 4(e)). EPSP activity in 40% hACSF ± NBQX closely followed the pattern established in 17% hACSF (Figure 4(f)). Blocking AMPA receptors reduced EPSPs by about 50% in all conditions. In sum, the observed excitability changes in 17% and 40% hACSF suggest that AMPA receptor activation is not required for the increase in APs evoked by hACSF, but AMPA receptors may play a larger role in contributing to the frequency of bursting activity as hypoosmolar conditions become more extreme.

**Figure 5.**
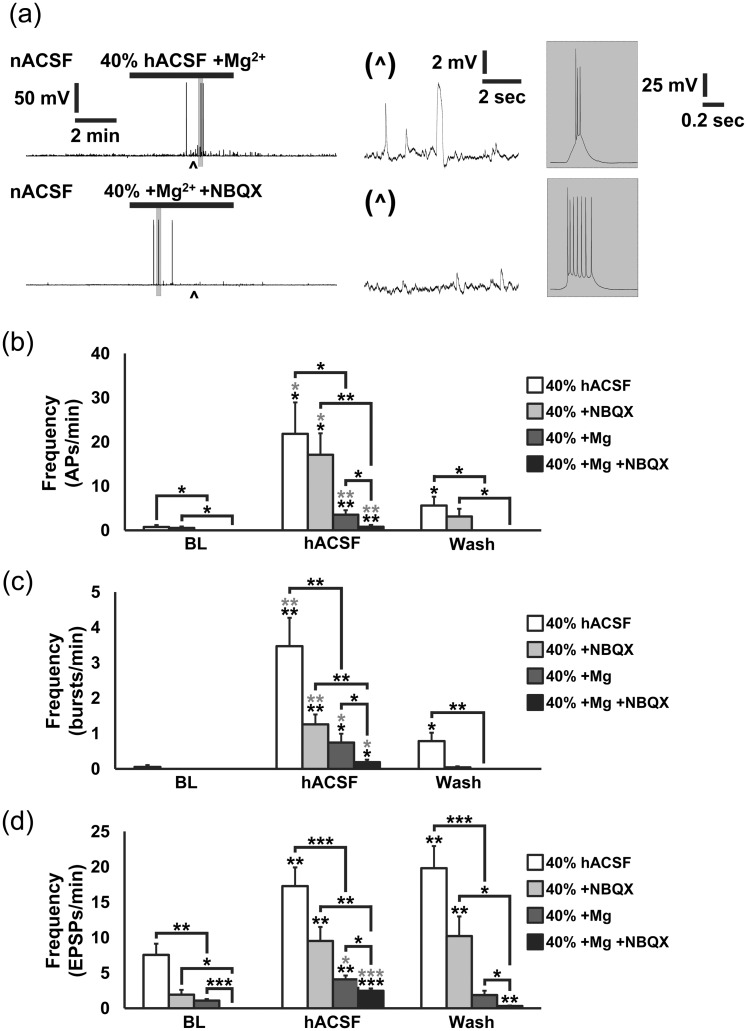
Neuronal action potentials and EPSPs evoked by hACSF in increasingly physiological conditions. (a) Representative current-clamp recordings of neuronal membrane potential in 40% hACSF with Mg^2+^ (diluted to ∼0.7 mM; upper traces) and 40% hACSF plus 10 µM NBQX (lower traces). Carets below each trace mark location of expanded EPSPs (middle panels), while shaded boxes mark location of expanded APs (right panels). (b) Frequency of total action potentials (single plus bursts) was significantly elevated over baseline (black asterisks) and wash periods (grey asterisks) in all conditions. Addition of Mg^2+^ reduced the frequency of APs overall, indicating the role for NMDARs. Note, however, that NMDARs were still activated significantly by hACSF with Mg^2+^ present. (c) Examination of bursts alone revealed very similar results as total APs. Bursting activity was significantly elevated over baseline in all conditions (black asterisks) and remained elevated compared with the wash period (gray asterisks). (d) Frequency of EPSPs was reduced in a stepwise fashion with addition of NBQX, Mg^2+^, and NBQX + Mg^2+^. Presence of Mg^2+^ alone had a greater effect on reducing EPSP frequency compared with addition of NBQX alone, indicated a greater role for NMDARs over AMPARs. (*p < .05, **p < .01, and ***p < .001) *n* = 8 to 10 cells per group.

Differential effects of NBQX on bursts of APs between 17% and 40% hACSF suggested that AMPA receptors influence neuronal excitability only at stronger doses of hACSF. However, AMPA receptors also normally play an important role in NMDA receptor activation, as they are the physiological means by which the synaptic membrane depolarizes sufficiently to remove magnesium block from the NMDA receptor channel. Since our nACSF and hACSFs contained 0 mM Mg^2+^, it could be argued that AMPA receptors play a more prominent role when Mg^2+^ is closer to physiological levels. To address this, we repeated the previous 40% hACSF experiments using nACSF containing physiological Mg^2+^ (1.3 mM), and hACSF made by dilution of the same. Inclusion of Mg^2+^ suppressed overall activity considerably (Figure 5(a)). However, application of hACSF ± NBQX still evoked sAPs, bAPs, and EPSPs in the presence of Mg^2+^ (Figure 5(a)). Compared with recordings in Mg^2+^-free hACSF (with or without NBQX), neurons in hACSF containing Mg^2+^ exhibited significantly fewer APs (Figure 5(b)) and bursts (Figure 5(c)). Action potentials occurring over the baseline and wash periods were completely abolished by the addition of Mg^2+^. As would be expected, Mg^2+^ reduced baseline EPSP activity by more than half of that observed in Mg^2+^-free hACSF or Mg^2+^-free hACSF + NBQX (Figure 5(d)). Inclusion of Mg^2+^ inhibited the increased EPSP frequency observed during the wash period. Frequency of EPSPs in Mg^2+^ was also suppressed by NBQX at all time points. Altogether, these data suggest that the relative contribution of AMPA receptors to hACSF-induced excitability is similar in the presence or absence of Mg^2+^, and that Mg^2+^ has a much more significant effect overall in reducing APs and bAPs than does NBQX. These findings point to a significant role for NMDA receptors in the generation of APs by osmotic edema, which we tested more specifically in subsequent experiments (see below).

### Neuronal Excitability Increases in hACSF are NMDA Receptor-Dependent and Predominantly Nonsynaptic

**Figure 6.**
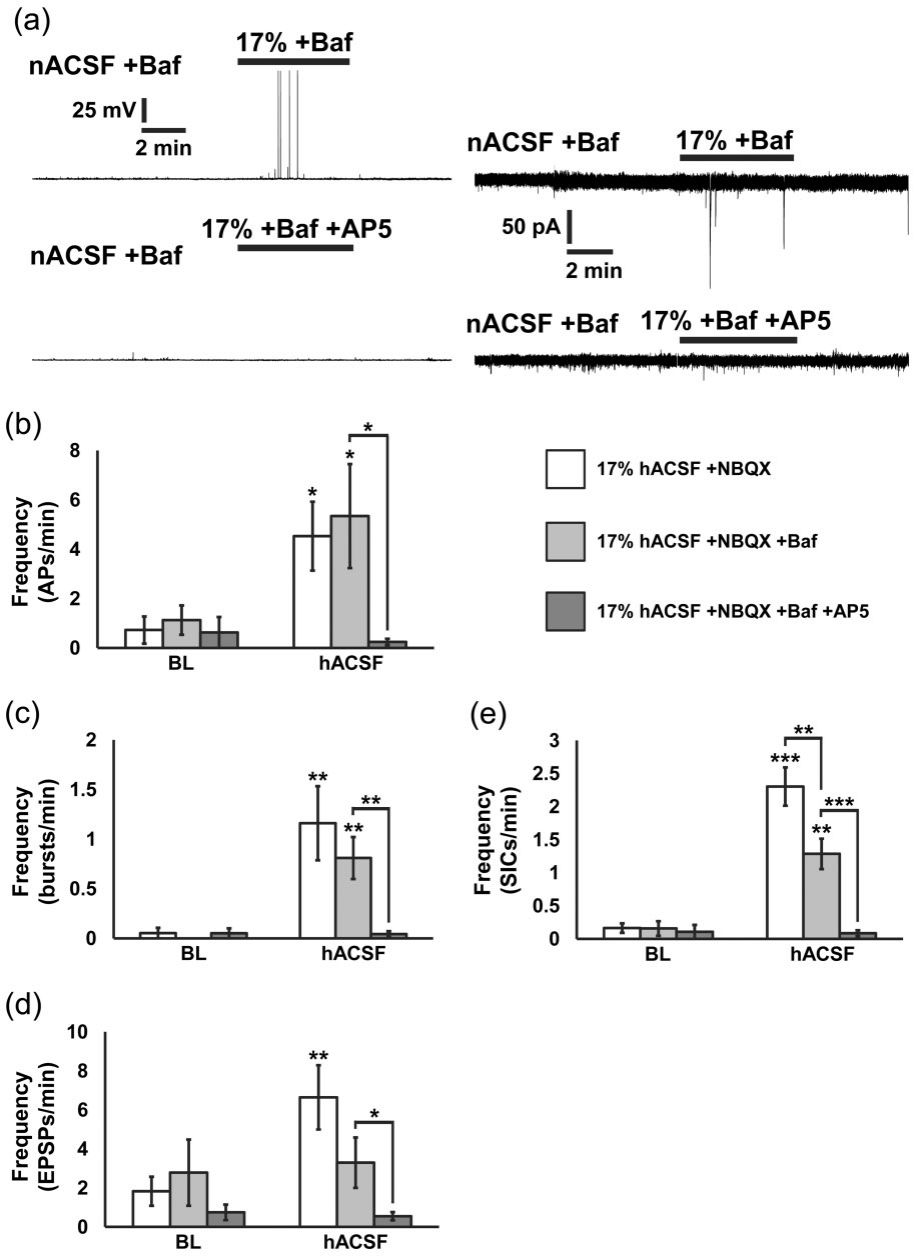
Action potentials and SICs evoked by osmotic edema are non-synaptic and NMDA receptor-dependent. (a) Current-clamp (left traces) and voltage-clamp (right traces) recordings of neuronal membrane potential and currents, respectively, in bafilomycin (upper panels) and bafilomycin (baf) + 50 µM AP5 (lower panels). NBQX and baf were present throughout. Application of 17% hACSF evoked SICs and APs independent of synaptic activity and which were blocked by the NMDA receptor antagonist. (b) Summary histogram indicating that APs remained significantly elevated on average in baf compared with baseline, and equal to the frequency of APs in hACSF without baf. AP5 nearly completely abolished APs. (c) Blockade of quantal vesicular release did not reduce bursting activity compared with bursts evoked with spontaneous vesicular release intact. Bursting activity was also completely blocked by AP5. (d) Not surprisingly, baf reduced the occurrence of EPSPs, which were no longer significantly elevated over baseline compared with the recordings performed without baf. AP5 significantly reduced the frequency of EPSPs, indicating that many of them are NMDA receptor-dependent. (e) Frequency of SICs was also significantly elevated non-synaptically but not quite to the extent observed with synaptic activity intact. Like APs, SICs were also nearly completely blocked by AP5. The data suggest that some portion of SICs may be due to activation of postsynaptic NMDARs. (*p < .05, **p < .01, and ***p < .001) *n* = 8 to 10 cells per group.

Swelling of brain tissue and subsequent reduction of the ECS can increase the ambient glutamate concentration, increasing the likelihood that nonsynaptic glutamate binds extrasynaptic receptors. Previous work has suggested that SICs evoked by various stimuli may be driven by nonsynaptic sources of glutamate (Angulo et al., 2004; Fellin et al., 2004; Kozlov et al., 2006; Fiacco et al., 2007). We chose to more closely examine how nonsynaptic sources of glutamate influence excitability of neurons by osmotic edema in our model. Synaptic transmission in our slices was abolished by incubating slices in Bafilomycin A1 (4 µM), an inhibitor of the vacuolar H^+^-ATPase responsible for loading synaptic vesicles, prior to recording activity in hACSF. Despite abolishing vesicular neurotransmitter release, excitability was still elevated by 17% hACSF + NBQX (Figure 6(a)). In fact, blocking synaptic transmission had no effect on the total frequency of APs (Figure 6(b)) or on the frequency of bAPs evoked by 17% hACSF (Figure 6(c)). Frequency of EPSPs was mildly but noticeably reduced by bafilomycin and was not significantly different from either 17% hACSF without bafilomycin, or its own baseline (Figure 6(d)). The most prominent effect of bafilomycin was on the frequency of SICs in hACSF, which was reduced by almost 50% (Figure 6(e)). In all cases, addition of the NMDA receptor antagonist DL-AP5 (50 µM) virtually eliminated the activity present in bafilomycin (APs and EPSPs, *p* < .05; bAPs, *p* < .01; SICs, *p* < .001), providing further evidence that NMDA receptor activation (but not vesicular glutamate release) is necessary for the observed hACSF-induced changes in neuronal excitability. However, the effect of bafilomycin on the frequency of SICs suggests that vesicular glutamate release may modulate SIC frequency or severity.

### SICs Induced by hACSF Are Enhanced by D-Serine and Blocked by Antagonists of NR2B-Containing NMDA Receptors

**Figure 7.**
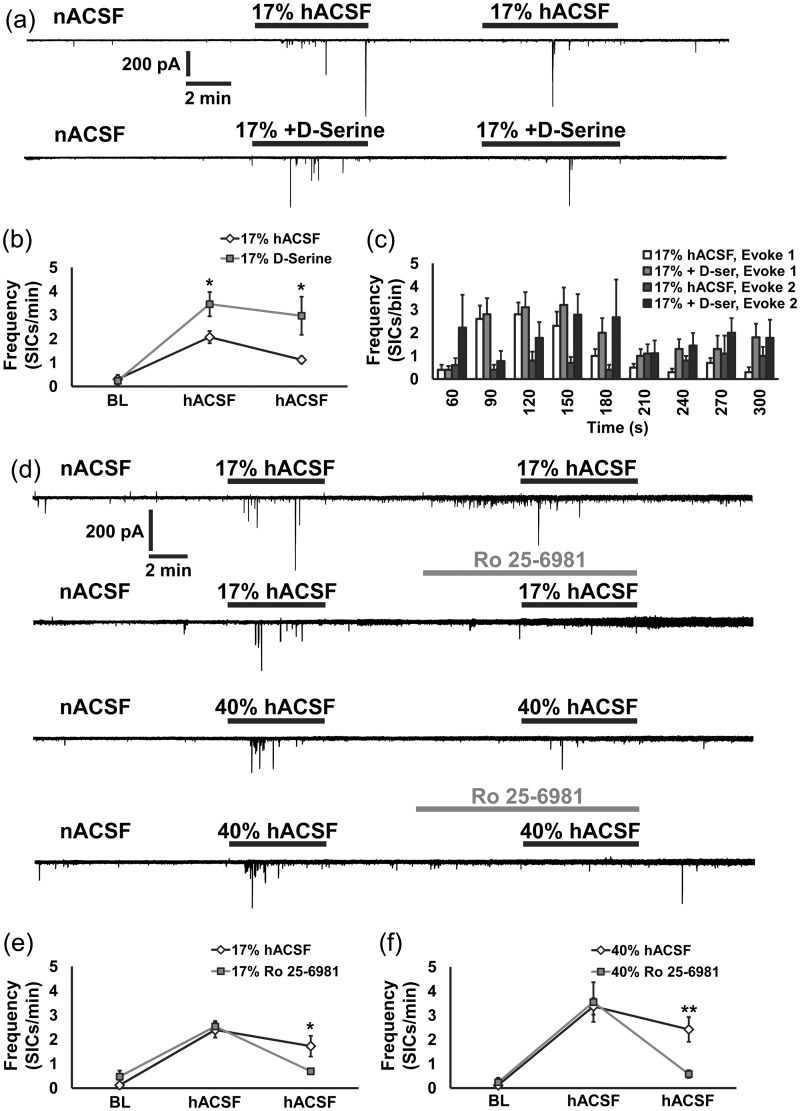
SICs are enhanced by D-serine and blocked by the NR2B subunit NMDAR antagonist Ro 25-6981. (a) Representative recordings of excitatory currents in CA1 pyramidal neurons before, during, and after 17% hACSF (upper trace) and 17% hACSF + D-serine (lower trace). In these experiments, D-serine was applied twice for easier comparison to within cell recordings in which NR2B NMDARs were blocked (see below). (b) On average, D-serine significantly enhanced the frequency of SICs during both hACSF applications compared with baseline and also compared with recordings in separate slices in which hACSF was reapplied without D-serine. (c) Frequency histogram of the hACSF period organized into 30 s bins revealed a right-ward shift in SIC frequency in the presence of D-serine (see 150 and 180 s bins). (d) Representative recordings of successive hACSF applications with or without the addition of 1 µM Ro 25-6981 for 17% hACSF (upper panels) or 40% hACSF (lower panels). Antagonism of NR2B subunit-containing NMDARs nearly completely blocked SICs evoked during osmotic challenge, as summarized in (e) and (f). (*p < .05 and **p < .01) *n* = 8 cells per group.

The near-complete block of SICs by DL-AP5 indicated that they were NMDA receptor-dependent. We substantiated this finding by recording SICs in 17% hACSF plus 10 µM D-serine, a required NMDA receptor co-agonist which binds to the glycine site on the NR1 subunit. D-serine concentrations are tightly regulated in vivo (Danysz and Parsons, 1998) but may be partially depleted by slice preparation. Therefore, elevating concentrations of D-serine to saturating levels in the slice is expected to potentiate NMDAR-dependent activity. Consistent with this, we observed a substantial increase in SIC frequency in slices bathed in 17% hACSF + D-serine (−D-serine: 2.07 ± 0.26 SICs/min; +D-serine: 3.46 ± 0.51 SICs/min; *p* < .05; Figure 7(a) and (b)). A second application of 17% hACSF without D-serine evoked SICs at a lower frequency than the first application (1.12 ± 0.15 SICs/min), whereas SIC frequency remained potentiated during the second application if D-serine was included, showing little to no attenuation (hACSF, 1.12 ± 0.15 SICs/min; D-ser, 2.97 ± 0.80 SICs/min; *p* < .05). Interestingly, while SICs occurred along a similar timecourse with or without D-serine during the first hACSF application, D-serine appeared to shift peak SIC production approximately 30 to 60 s later during the second hACSF application (Figure 7(c)).

Exogenous D-serine has been shown to specifically enhance activity of NR2B-containing NMDA receptors (Duffy et al., 2008). SICs are identified in part by their slow rise and decay times, which may partially result from the kinetics of the particular NMDA subtypes activated. Previous work has suggested that SICs (resulting from different manipulations) are mediated by NMDA receptors containing the NR2B subunit (Fellin et al., 2004). Using the highly selective NR2B antagonist Ro 25-6981 (1 µM), we investigated whether SICs in hACSF were similarly dependent on the NR2B subunit (Figure 7(d)). As in the previous experiment, SICs were evoked successively using repeated applications of 17% (top traces) or 40% hACSF (bottom traces). SICs were almost completely abolished by the addition of Ro 25-6981 in 17% or 40% hACSF (Figure 7(d) and (e)). These data indicate that NR2B subunit-containing NMDA receptors play a critical role in the increased neuronal excitability produced by acute cellular edema.

### Neuronal Excitability Evoked by hACSF Is More Pronounced in Adult Compared With Juvenile Mice

**Figure 8.**
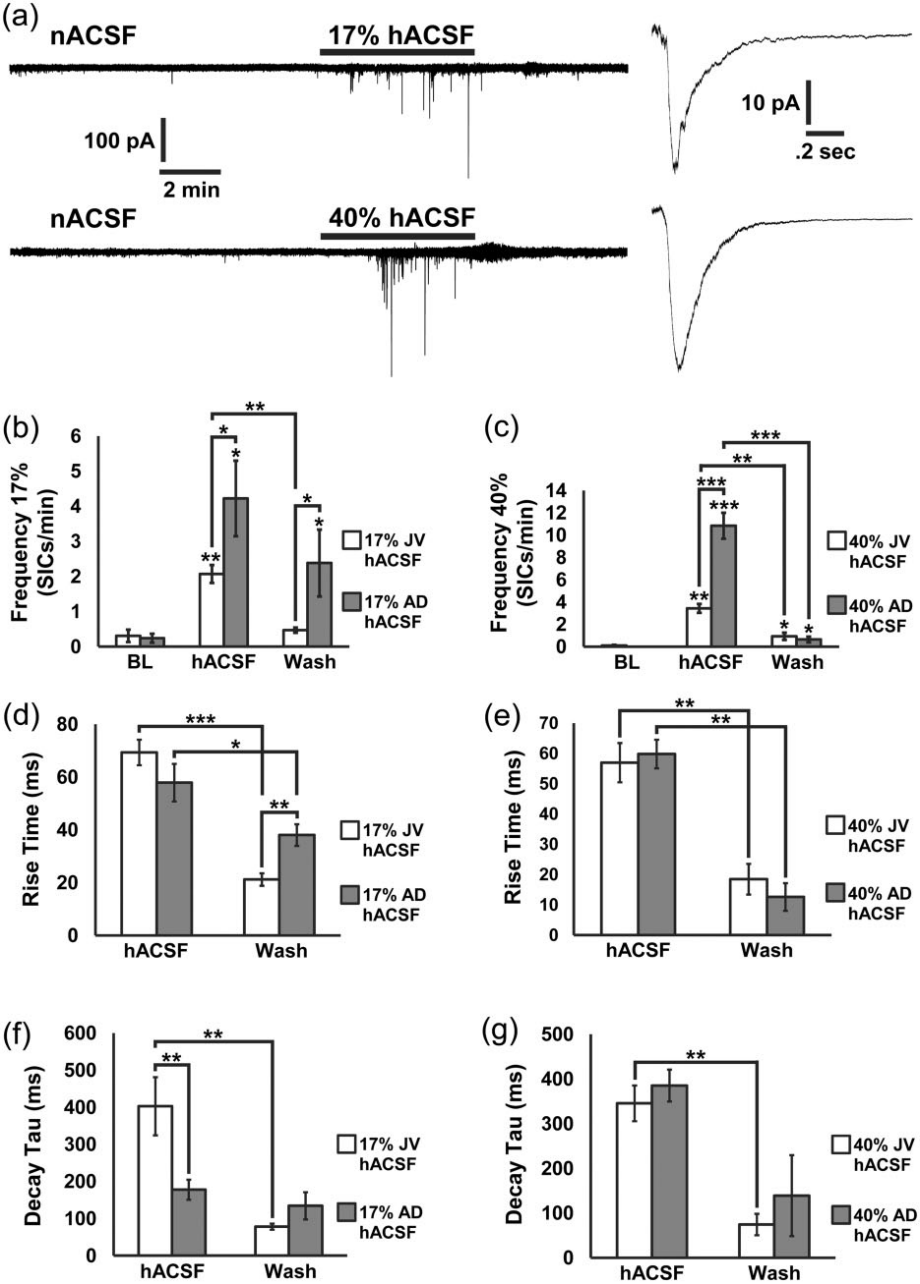
Neuronal excitability is elevated to a greater extent in adult versus juvenile hippocampus. (a) Representative recordings of neuronal SICs in hippocampal slices from 8 to 12-week-old adult mice before and during application of 17% hACSF (upper trace) or 40% hACSF (lower trace). To the right of each recording, the averaged SIC is shown for the hACSF period. (b, c) Frequency of SICs evoked by 17% and 40% hACSF was significantly higher in adult mice compared with juveniles. SICs remained elevated longer during the wash period in 17% hACSF in adults versus juvenile mice (b). (d, e) Rise times of SICs occurring during osmotic challenge were significantly slower in 17% (d) and 40% hACSF (e) for both juvenile and adult mice compared with SICs occurring after return to nACSF (“wash”). Too few SICs occurred during the baseline period for comparison. (f) Decay taus of SICs evoked by 17% hACSF were significantly shorter in adult tissue compared with juvenile, and not significantly different compared with the wash period. (g) Decay taus of SICs evoked by 40% hACSF were similar to those evoked in juvenile slices. (*p < .05, **p < .01, and ***p < .001) *n* = 8 for 17% hACSF; *n* = 9 for 40% hACSF.

Experiments in hippocampal slices from mouse pups provide useful information about cerebral edema and epilepsies in juveniles, but data may not be applicable to adults. Numerous differences exist between adult and juvenile brain tissue, including degree of synaptic development, function, and expression levels of receptors, and regulation of neurotransmitter concentrations and reuptake. Furthermore, interstitial space is smaller in adult tissue compared with juvenile tissue (Lehmenkuhler et al., 1993; Kilb et al., 2006), suggesting that effects of cellular edema may be quite different between different age groups. To explore this further, we next performed experiments using hippocampal slices obtained from 8 to 12-week-old adult mice. As observed in juveniles, adult neurons exhibited a marked increase in SIC activity at both 17% (Figure 8(a), top) and 40% (bottom) hACSF doses, while activity over the baseline period prior to hACSF application was rare or nonexistent in most cells (Figure 8(b); baseline participation 3/8 cells). Compared with juveniles, however, adults had more than double the number of SICs/minute during 17% hACSF application (JV 2.07 ± 0.26 SICs/min, AD 4.22 ± 1.08 SICs/min; *p* < .05), and in the wash period (JV 0.47 ± 0.07 SICs/min, AD 2.38 ± 0.95 SICs/min; *p* < .05). Further emphasizing the heightened excitability of adult tissue, frequency of SICs in adults exposed to 40% hACSF was over 3 times higher than in juveniles (Figure 8(c); JV 3.44 ± 0.39 SICs/min, AD 10.85 ± 1.16 SICs/min; *p* < 0.001). Curiously, the frequency of SICs in adults in the wash period after 40% hACSF was not significantly different from juveniles, despite the pronounced elevation in the wash period following 17% hACSF. Rise time of SICs (Figure 8(d)) differed between juveniles and adults only during the wash period following 17% hACSF (JV 21.18 ± 2.37 ms, AD 38.03 ± 4.09 ms; *p* < .01). Adult SICs also decayed significantly faster than juvenile during 17% hACSF application (Figure 8(f); 402.75 ± 78.26 ms juvenile, 177.82 ± 27.07 ms adult, *p* < .01). Neither rise time nor decay tau differed between juveniles and adults in 40% hACSF (Figure 8(e) and (g)), suggesting that intrinsic properties of SICs do not change between the age groups. Rather, the kinetic differences in 17% hACSF treatment groups are likely driven by age-dependent differences in the ECS and tissue swelling characteristics.

## Discussion

Our results demonstrate that one of the very first effects produced in CA1 pyramidal neurons by osmotic edema are NMDA receptor-dependent SICs. SICs were generated within about 60 s in hACSF at all concentrations tested. Even 5% reduction in osmolarity evoked SICs in neurons, although SICs were larger and more frequent as osmolarity decreased. SICs are typically evoked in Mg^2+^-free conditions or depolarized holding potentials, during voltage-clamp of neurons, and in the presence of the NMDA receptor co-agonist D-serine, the AMPA receptor antagonist NBQX, and in TTX or bafilomycin to block APs and quantal vesicular release (Angulo et al., 2004; Fellin et al., 2004; Fiacco et al., 2007; Shigetomi et al., 2008). These conditions make it difficult to ascertain what effect SICs might have on neuronal excitability under more physiological conditions. Therefore, the effect of hypoosmolarity was measured while systematically removing pharmacological and electrophysiological manipulations. In all cases tested, including in the absence of any added reagents or voltage clamp, reducing osmolarity of the ACSF significantly increased neuronal excitability. APs were evoked over the same timecourse as SICs and were also blocked by AP5, suggesting that activation of NMDA receptors during cell swelling significantly depolarizes neurons above AP threshold. APs also displayed more bursting characteristics during osmotic challenge compared with the few APs occurring spontaneously in the baseline period prior to hACSF application. Last, we found that neuronal excitability was more pronounced in adult versus juvenile tissue. Our results suggest that significant increases in neuronal excitability are produced at the very onset of cell swelling, likely before cellular volume increases have stabilized.

Previous work recorded the effects of reduced osmolarity on evoked neuronal activity in the hippocampus. Andrew et al. (1989) and Ballyk et al. (1991) observed effects of hypoosmolality on evoked CA1 pyramidal cell field potentials and on the PS amplitude, with no evidence for changes in chemical synaptic transmission. Because the increase in PS amplitude was similar when antidromically stimulating CA1 axons or orthodromic stimulation of Schaffer collaterals, the changes observed were entirely explained by the increased extracellular resistance directing more voltage across the neuronal membrane, as described by Ohm’s law (voltage = current × resistance; Andrew et al., 1989). These ephaptic or field effects help to synchronize neuronal excitability across the population of neurons and, therefore, contribute to seizure-like discharges (Andrew et al., 1989; Ballyk et al., 1991). Similar results were obtained from recordings in CA3 by the same group (Saly and Andrew, 1993). Later, Somjen and others challenged these findings by providing evidence from recording evoked potentials and whole-cell synaptic currents that synaptic transmission is also enhanced by lowered osmolarity ACSF (Chebabo et al., 1995a; Huang et al., 1997). These authors hypothesized that buildup of transmitter concentration in the reduced ECS at the receptive surface may be the mechanism underlying the increased EPSCs. Together, these seminal studies provided strong evidence that osmotically induced edema enhances evoked synaptic transmission, electrical field effects, and population discharges. However, none of these earlier studies mentioned SICs or found any changes in electrophysiological properties of individual neurons. It now seems likely that this activity was missed in the previous work due to the methods used to record neuronal activity. Episodic rather than continuous recordings were performed, usually after several minutes (10–20 min) in hACSF when cell swelling and reduction of the ECS had largely stabilized. In contrast, we performed continuous recordings of spontaneous neuronal activity throughout the process of solution exchange from nACSF to hACSF. Our findings, therefore, add to and expand upon these earlier studies by identifying NMDA receptor activation and SICs as one of the first events that significantly increase neuronal excitability at the onset of osmotic edema.

SICs have been predominantly discussed in the context of astrocytes. The first reports of SICs suggested that astrocyte Ca^2+^-dependent glutamate release activated extrasynaptic NMDA receptors on adjacent neuronal compartments to generate SICs (Angulo et al., 2004; Fellin et al., 2004). However, these findings were later questioned by data suggesting that reduced osmolarity and cellular swelling were important for SICs. First, Kozlov et al. (2006) found that SICs in olfactory bulb granule cells were evoked by various types of mechanical stimulation including cyclic stretch to blood vessels, and that reducing extracellular osmolarity significantly increased SIC frequency. These data suggested a role for volume-regulated anion channels (VRAC) in the generation of SICs (Kozlov et al., 2006). Subsequently, Fiacco et al. (2007) demonstrated that SICs could be evoked in CA1 pyramidal neurons by application of −25 mM NaCl ACSF in slices from IP_3_R2^−/−^ mice, in which Gq GPCR-driven astrocyte Ca^2+^ activity is significantly reduced (Petravicz et al., 2008; Srinivasan et al., 2015). These findings suggested that SICs are predominantly driven by reduced osmolarity, although there is ample evidence to suggest that astrocyte Ca^2+^ activity amplifies or modulates volume-regulated release pathways that may be involved in the generation of SICs (Mongin and Kimelberg, 2005; Takano et al., 2005; Ramos-Mandujano et al., 2007).

**Figure 9.**
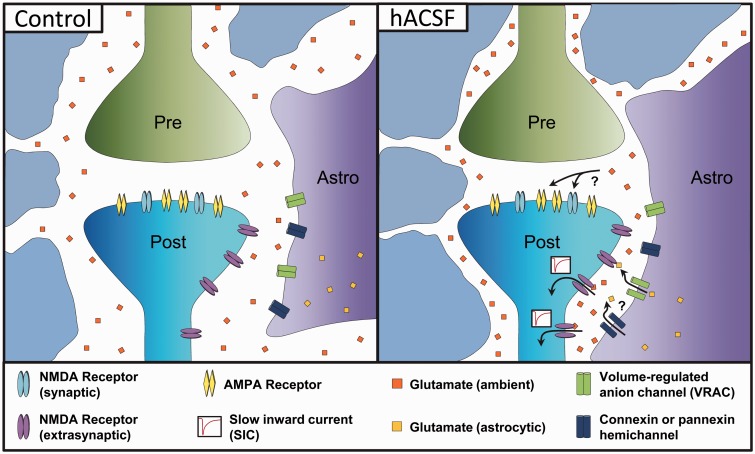
Hypothesized model for neuronal excitability increases in hACSF. In control conditions (left panel), ambient glutamate diffuses through the extracellular space around the synapse at low concentration. Neuronal excitability is mostly synaptic. Upon addition of hACSF (right panel), tissue (including astrocyte) swelling leads to constriction of the ECS. This increases local glutamate concentrations near extrasynaptic (and possibly synaptic) NMDA receptors, increasing neuronal excitability through NMDA receptor activation. Resultant currents (SICs) are large and slow due in part to the restricted diffusion of glutamate within the compressed ECS. Ambient glutamate concentration may be augmented by astrocytic glutamate released through volume-regulated anion channels (VRAC) and connexin or pannexin hemichannels.

Are astrocytes responsible for SICs? This remains an open question that requires further study. A hypothesis for how SICs are generated during the onset of cellular edema can be made after taking into consideration the cells that swell, the receptors involved, and where the glutamate is coming from (Figure 9). Evidence to date suggests that neurons are very resistant to osmotic changes. This has been demonstrated in acutely dissociated neurons (Somjen et al., 1993) and in recordings of real-time volume responses during acute osmotic stress in situ (Andrew et al., 2007) and in vivo (Risher et al., 2009). On the contrary, astrocytes readily swell in hypoosmolar conditions. Astrocyte swelling during osmotic edema has been attributed to their selective expression of the functional water channel aquaporin 4 (Andrew et al., 2007; Hirrlinger et al., 2008; Risher et al., 2009). Therefore, astrocytes appear to be the cells in the brain that swell in cellular edema brought on by acute reductions in extracellular osmolarity and may, therefore, be the driving force behind reduction of the ECS.

All studies of SICs, regardless of proposed mechanism, have found that SICs are mediated by NMDA receptors. In the present study, we also observed that NMDA receptors are responsible for SICs. SICs were potentiated by D-serine, and blocked by both DL-AP5 and the NR2B specific compound Ro25-6981. Previous work postulated that extrasynaptic NMDA receptors generate SICs because of their slow kinetics and block by NR2B subunit-specific antagonists such as ifenprodil (Fellin et al., 2004; Araque et al., 2014). However, accumulating evidence suggests that synaptic and extrasynaptic NMDA receptors are triheteromeric, containing GluN1, GluN2A (NR2A), and GluN2B (NR2B) subunits (Harris and Pettit, 2007; Rauner and Kohr, 2011; Tovar et al., 2013). Therefore, subunit selective pharmacology cannot be used to distinguish between synaptic versus extrasynaptic pools of NMDA receptors. If receptor composition is similar within and outside the synapse, kinetic properties of the channels themselves are not likely to play much of a role in the slow kinetics of SICs. Whether synaptic or extrasynaptic, a more likely scenario for the slow rise and decay times of SICs is the increased tortuosity of the ECS brought about by cell swelling. The ECS is not simply a fluid-filled space, but rather contains a rich meshwork of extracellular matrix molecules (Vargova and Sykova, 2014). The ECS can be likened to a wet sponge, which gets compressed when cells swell. Within this collapsed meshwork, diffusion of glutamate will be significantly impeded, leading to currents with slow kinetics. While a role for extrasynaptic NMDA receptors in the generation of SICs still seems likely based on their location and high-binding affinity for glutamate, additional work is required to substantiate this possibility. Incorporation of stimulation protocols with the use-dependent channel blocker MK-801 seems like a logical approach to isolate the extrasynaptic fraction of NMDA receptors (Harris and Pettit, 2007).

Because SICs can be evoked in the absence of neuronal APs or miniature spontaneous vesicular release, the glutamate binding NMDA receptors must be predominantly nonsynaptic in origin. This leaves two possible sources of glutamate leading to SICs: release from non-neuronal cells or ambient glutamate already present in the ECS which increases in concentration and proximity to receptors as the ECS shrinks. Astrocytes represent a potentially significant source of glutamate when they swell. It is well established that astrocytes in vitro release large quantities of glutamate from VRAC in hypoosmolar conditions (Abdullaev et al., 2006; Kimelberg et al., 2006; Liu et al., 2006). However, there is some uncertainty in the literature with regard to when or how VRAC open in the process of osmotic edema. Purified astrocytes in culture initially swell in response to hypoosmolar treatment and then undergo a regulatory volume decrease (RVD) due to opening of VRAC (Kimelberg et al., 2006; Andrew et al., 2007). VRAC opening causes compensatory loss of K^+^, Cl^−^, and excitatory amino acids including glutamate, accompanied by water, leading to recovery of cell volume. However, a number of studies have provided evidence that RVD does not occur in intact tissue preparations, as demonstrated by recording intrinsic optical signals (Andrew et al., 1997; Andrew et al., 2007) and real-time volume measurements of fluorescently labeled astrocytes in situ (Andrew et al., 2007; Hirrlinger et al., 2008) and in vivo (Risher et al., 2009). In few instances when RVD has been observed, it occurs gradually, proceeding over tens of minutes and does not appear to initiate for approximately 10 to 20 min in hypoosmolar solution (Chebabo et al., 1995a, 1995b; Andrew et al., 1997). In the present study, hACSF was only applied for 5 min. Our real-time volume measurements of astrocytes indicate that astrocyte volume increases are just starting to peak after 5 min in hACSF. This means that SICs and APs occurred over a period of time in which the ECS was still shrinking, and nACSF was reapplied before RVD had a chance to begin. Previous studies recording changes in light transmittance and extracellular resistance measurements also suggest that cell swelling and reduction of the ECS is still occurring during this time (Andrew and MacVicar, 1994; Chebabo et al., 1995b; Kilb et al., 2006; Andrew et al., 2007). Initiation of SICs within 1 min suggests—at least in the context of RVD measurements—that sufficient glutamate is available to activate NMDARs before VRAC ever have a chance to open. If, however, VRAC are triggered to open by the initial stretch of the membrane as the cell volume is increasing, they may play a role. Based on measurements of glutamate accumulation in the ECS by enzymatic assay and D-[^3^H]aspartate release in vitro, and microdialysis measurements in vivo, significant release of glutamate from VRAC takes place within 3 to 5 min, likely while cells are still swelling (Abdullaev et al., 2006; Liu et al., 2006; Haskew-Layton et al., 2008). Alternatively, other mechanically sensitive channels may be involved in generation of SICs. The NMDA receptors themselves have been reported to be osmosensitive (Paoletti and Ascher, 1994), and connexin or pannexin hemichannels are sensitive to both stretch and reduced divalent cation solution (Ye et al., 2003; Montero and Orellana, 2015; Voigt et al., 2015)—precisely the conditions presented by hypoosmolar challenge. Further complicating matters is that the traditional pharmacological agents used to block VRAC are nonspecific, including the most promising of those—DCPIB—which has been shown recently to also block hemichannels (Bowens et al., 2013). At this time, it is not possible to determine if SICs are generated by an active release mechanism or by glutamate already present in the ECS. A careful series of experiments will need to be performed to determine the role of VRAC or other channels in the generation of SICs and increased neuronal excitability during acute osmotic edema. An intriguing possibility is that hemichannels release glutamate in the early phase while cell volume is increasing, while VRAC are involved in sustained release of glutamate during prolonged RVD.

Our findings have important implications for understanding underpinnings of epilepsy. Nonsynaptic mechanisms are critical for the initiation and maintenance of recurring seizure-like activity in the hippocampus. Ictal discharges have actually been shown to occur in the complete absence of chemical synaptic transmission (Jensen and Yaari, 1988; Dudek et al., 1990; Roper et al., 1992). Undoubtedly, increased electrical field effects due to reduction of the ECS play a major role in synchronization of neuronal bursts underlying seizure (Taylor and Dudek, 1984; Andrew et al., 1989; Jensen and Yaari, 1997; Dudek et al., 1999). Chemical synaptic transmission has also been shown to play a role (Huang et al., 1997), likely amplifying seizure activity through activation of excitatory AMPA and NMDA receptors to increase neuronal depolarization. Dingledine et al. (1986) found that a significant component of the slow depolarization underlying burst firing is synaptic in origin and mediated by NMDA receptors. Later, Traynelis and Dingledine (1988) demonstrated that recurring potassium-induced hippocampal seizures (ictal discharges) are blocked by the NMDA receptor antagonist D-AP5. SICs are intriguing in the context of epilepsy, as they are nonsynaptic in origin, chemically mediated, and NMDA receptor dependent. SICs enhance synchrony of neuronal activity, as demonstrated in the present study by increased bursting of neuronal APs evoked by hACSF application, an effect that was dependent on NMDA receptors. Our work provides proof-of-principle that rapid cell swelling can increase and synchronize neuronal excitability in juvenile and adult hippocampus through the generation of SICs. The greater effect observed in adult hippocampal slices may be due to the smaller ECS in adults, which is estimated to be about half the size of juveniles (Kilb et al., 2006). Overall, the consistent effect of osmotic edema on neuronal excitability across a variety of conditions suggests that cell swelling may be a universal mechanism contributing to seizures across multiple forms of epilepsy and seizure disorders. The rapid timecourse of evoking SICs by hACSF in our study fits well with the timing of glial depolarization, increased CA1 burst intensity, and ECS reduction, which occur 1 to 2 min prior to ictal discharge in the elevated potassium model of epilepsy (Traynelis and Dingledine, 1988, 1989). Preseizure ECS constriction (Binder et al., 2004) and tissue impedance changes (Olsson et al., 2006; Broberg et al., 2008) have also been observed in vivo. It may be that elevated potassium, which has also been shown cause cell swelling (Andrew et al., 2007; Risher et al., 2009), leads to opening of VRAC, RVD, and release of glutamate to trigger the NMDA receptor-dependent ictal discharge.
